# Profiles of Serum Cytokines in Acute Drug-Induced Liver Injury and Their Prognostic Significance

**DOI:** 10.1371/journal.pone.0081974

**Published:** 2013-12-27

**Authors:** Nury M. Steuerwald, David M. Foureau, H. James Norton, Jie Zhou, Judith C. Parsons, Naga Chalasani, Robert J. Fontana, Paul B. Watkins, William M. Lee, K. Rajender Reddy, Andrew Stolz, Jayant Talwalkar, Timothy Davern, Dhanonjoy Saha, Lauren N. Bell, Huiman Barnhart, Jiezhun Gu, Jose Serrano, Herbert L. Bonkovsky

**Affiliations:** 1 The Liver-Biliary-Pancreatic Center and Departments of Internal Medicine, General Surgery, Research, and Dickson Advanced Analytics, Carolinas Medical Center, Charlotte, North Carolina, United States of America; 2 Department of Internal Medicine, IUPUI, Indianapolis, Indiana, United States of America; 3 Department of Internal Medicine, University of Michigan, Ann Arbor, Michigan, United States of America; 4 Schools of Medicine and Pharmacy, University of North Carolina, Chapel Hill, North Carolina, United States of America; 5 Department of Internal Medicine, University of Texas Southwestern Medical Center, Dallas, Texas, United States of America; 6 Department of Internal Medicine, University of Pennsylvania, Philadelphia, Pennsylvania, United States of America; 7 Department of Internal Medicine, University of Southern California, Los Angeles, California, United States of America; 8 Department of Internal Medicine, Mayo Clinic, Rochester, Minnesota, United States of America; 9 Department of Internal Medicine, Pacific Medical Center, San Francisco, California, United States of America; 10 Duke Clinical Research Institute, Durham, North Carolina, United States of America; 11 National Institute of Diabetes and Digestive and Kidney Diseases, NIH, Bethesda, Maryland, United States of America; University of Montreal, Canada

## Abstract

Drug-induced liver injury (DILI) is the most common cause of acute liver failure in the United-States. The aim of the study was to describe serum immune profiles associated with acute DILI, to investigate whether there are profiles associated with clinical features or types of DILI and/or with prognosis, and to assess temporal changes in levels. Twenty-seven immune analytes were measured in the sera of 78 DILI subjects in the Drug-Induced Liver Injury Network (DILIN) and compared with 40 healthy controls. Immune analytes (14 cytokines, 7 chemokines and 6 growth factors) were measured by BioPlex multiplex ELISA at DILI onset and after 6 months. A modeling process utilizing immune principles was used to select a final set of variables among 27 immune analytes and several additional clinical lab values for prediction of early death (within 6 months of DILI onset). Nineteen of the 27 immune analytes were differentially expressed among healthy control, DILI onset and 6-month cohorts. Disparate patterns of immune responses, especially innate and adaptive cellular (mostly TH17) immunity were evident. Low values of four immune analytes (IL-9, IL-17, PDGF-bb and RANTES) and serum albumin are predictive of early death [PPV = 88% (95% CI, 65%-100%), NPV = 97% (95% CI, 93%-100%), accuracy = 96% (95% CI, 92%-100%)].

**Conclusions:**

Acute DILI is associated with robust and varying immune responses. High levels of expression of cytokines associated with innate immunity are associated with a poor prognosis, whereas high levels of expression of adaptive cytokines are associated with good long-term prognosis and eventual recovery. Serum immune analyte profiles at DILI onset appear to be of prognostic, and perhaps, diagnostic significance.

## Introduction

Liver injury caused by drugs and chemicals is a problem that continues to grow in prevalence and importance. Drug induced liver injury (DILI) is a common cause of acute liver failure in the United States [1-3]. DILI is also a frequent adverse drug event that leads to the abandonment of otherwise promising new drug candidates or to the withdrawal from the market of new drugs [4,5].

There are many challenges to making a clear diagnosis of DILI, because there is no pathognomonic test or “gold standard” for establishing the diagnosis. Rather, it is usually a diagnosis of exclusion, after compatible history of drug exposure has been elicited and other more frequent or likely causes have been excluded [6,7]. The problem is compounded by the growing use of herbal remedies and dietary supplements (HDS), which account for ~10% of instances of DILI in the USA [8] and even higher frequencies in the Far East [9]. Furthermore, there are difficulties in predicting the prognosis of acute DILI. Levels of the most commonly used biomarkers, (e.g. serum aminotransferases, alkaline phosphatase, or other enzymes) are of limited sensitivity and specificity [7,10]. Addition of serum total bilirubin, especially in hepatitis-type injury with increases chiefly in serum aminotransferases, is useful, as was emphasized by Zimmerman more than 30 years ago. Clinically apparent jaundice with elevated levels of serum total bilirubin and high levels of serum aminotransferases due to drugs are ominous, with about 10% of subjects succumbing during the acute phase of illness [11]. Better biomarkers and early warning signals of serious, potentially fatal DILI are urgently needed [5,10].

Changes in levels of circulating cytokines and chemokines have been proposed as possible biomarkers of tissue injury, including liver injury due to drugs [5]. Indeed, several cytokines [12-16], and individual or small groups of cytokines have been reported to be altered in a few experimental studies of DILI [17-21]. For example, in one study in humans, genetic polymorphisms associated with lower production of IL-10, were associated with lower eosinophil counts and poorer outcomes [13].

The aim of this study was to describe serum immune profiles associated with acute DILI, to investigate whether there are profiles associated with clinical features or types of DILI and/or with prognosis, and to assess temporal changes in levels of the analytes. To achieve these aims, we simultaneously measured levels of 27 immune analytes in the sera of subjects with well-characterized, carefully studied, acute DILI, who were then followed for at least one year in the prospective US Drug-Induced Liver Injury Network. This Network and its major methods have been described previously [8,22,23], and findings in the first three hundred subjects enrolled have been described [8]. In this work, we compared results from 78 subjects with acute DILI, with those of 40 normal controls in whom serum proteomic profiles were reported recently [24]. We observed striking changes in innate and adaptive cellular responses, and we also developed a new means for early prediction of outcomes, based upon results at initial acute presentation for four serum immune analytes (interleukin[IL] 9, IL 17, platelet derived growth factor bb, and RANTES) and the level of the serum albumin. Some of these results have been presented in abstract form [25].

## Materials and Methods

### Subjects studied

Serum samples from subjects with DILI were collected as part of the Drug Induced Liver Injury Network (DILIN) prospective study, in which subjects with suspected DILI were enrolled and detailed clinical data were collected [22]. Written informed consent was obtained at that time. Clinical data were reviewed by the DILIN Causality Committee, which made the final determination as to whether the case qualified as *bona fide* DILI, and assigned each implicated drug a probability of having caused DILI [23]. Types of liver injury and R-values were as described [8,24]. We studied 78 subjects with acute DILI, enrolled between December 2004 and July 2010, and who had serum samples taken within two weeks of clinical onset. These were the same subjects whose serum proteomic profiles were described by Bell et al [24]. The study protocol was approved by the Institutional Review Board of Indiana University-Purdue University, Indianapolis, Indiana (Dr. Naga Chalasani as PI). Whenever possible, subjects were followed for at least 6 months to assess whether there was recovery or evidence of development of chronic DILI. Blood samples were collected and sent to the central DILIN sample repository for processing and storage at -80°C. Sera from 40 healthy controls (volunteer blood donors) were obtained from a blood bank [24]. These controls had no history or evidence of liver disease, had normal serum levels of ALT, AST, AP, and total serum bilirubin, and had no serological evidence of active HAV, HBV, HCV, or HIV infection. 

### Immune analyte profiling by Bio-Plex assay

Concentrations of immune analytes in sera were determined using a human 27-plex assay [14 cytokines (IL-1β, IL-1ra, IL-2, IL-4, IL-5, IL-6, IL-9, IL-10, IL-12, IL-13, IL-15, IL-17, IFN-γ, TNF-α); 7 chemokines (Eotaxin, IL-8, IP-10, MCP-1, MIP-1α, MIP-1β, RANTES); and 6 growth factors (IL-7, FGF basic, G-CSF, GM-CSF, PDGF-BB, VEGF)] (Bio-Plex Suspension Array System, Bio-Rad, Hercules, CA, USA), following the manufacturer’s instructions. Samples were diluted 1:4 (*v:v*) in sample diluent and incubated for 30 minutes (room temperature, 300 rpm agitation) with capture antibody-coupled magnetic beads. Following three washes in a Bio-Plex Pro wash station, samples were incubated for 30 minutes in the dark (room temperature, 300 rpm agitation) with biotinylated detection antibody. Each captured analyte was detected by the addition of streptavidin-phycoerythrin and quantified using a BioPlex array reader. Analyte concentrations were calculated with Bio-Plex Manager software. 

### Heat-maps of immune analytes in DILI subjects

**Figure 1:**
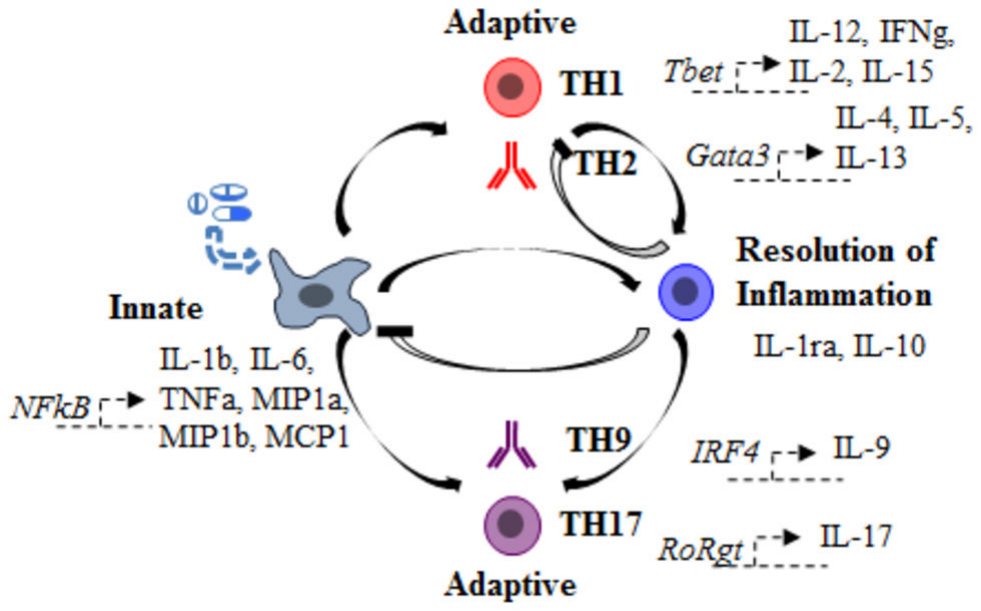
Simplified model of immune responses. Immune stimuli trigger NFκB nuclear translocation and early/innate cytokine production (IL-1β, IL-6, TNF-α) by damaged tissue. If this early inflammatory state persists, it will activate adaptive immune processes favoring either cellular (T-box transcription factor TBX21 (e.g., T-bet)-dependent / TH1-type: IL-12p70, IFNγ, IL-2, IL-15) or humoral (Gata3-dependent /TH2-type: IL-4, IL-5, IL-13) responses. This inflammatory state may ultimately resolve itself by triggering anti-inflammatory processes (IL-10, IL-1ra). In the event of an unresolved inflammatory state, innate and anti-inflammatory processes synergize to evolve into RoRγt-dependent / TH17-type: IL-17 or IRF4-dependent TH9-type IL-9 adaptive immunity.

 To generate cytokine heat-maps that were used to establish and compare immune response patterns among DILI subjects, we grouped cytokines, based on the major known transcription factors that trigger their expression and the immune processes to which they primarily relate, as follows: cytokines associated with innate immunity (NFκB-dependent): IL-1β, IL-6, TNFα; cytokines associated with adaptive cellular immunity (T-box transcription factor TBX21/T-bet or RoRγt-dependent): IL-12p70, IFNγ, IL-2, IL-15, IL-17; cytokines associated with adaptive humoral immunity (Gata3 or IRF-4-dependent): IL-4, IL-5, IL-13, IL-9; and cytokines associated with immuno-suppression / resolution of inflammation: IL-1ra, IL-10 [26-30] (Figure **1**).

Normal ranges for serum concentrations of immune analytes were based on measurements obtained from the healthy controls and recorded as mean ± 1 standard deviation (SD). Concentrations in sera of DILI subjects 1 SD higher or lower than the means of healthy subjects were defined as abnormal. We identified four main immune profiles, based on patterns of abnormal cytokine expression among the DILI cohort at onset: 1) DILI of “mixed-immune” profile in which at least 1 innate and 1 or more adaptive cellular and humoral cytokine concentrations were higher than normal; 2) DILI of “innate immune” profile in which 1 or more innate cytokine concentrations were higher than normal and no adaptive cytokine was higher than normal; 3) DILI of “adaptive immune” profile in which 1 or more adaptive cellular or humoral cytokine concentrations were higher than normal; and 4) DILI of “normal-immune” profile with no abnormal innate cytokine and at most one abnormal adaptive cytokine concentrations. DILI immune profiles that did not match any of the criteria described above were labeled as “uncategorized.”

### Statistical Methods

Descriptive statistics, including means and standard deviations, or counts and percentages were calculated. For data measured on the interval scale, the Student’s t-test or analysis of variance (ANOVA) was used. If the data were not normally distributed, the Wilcoxon rank sum test or the Kruskal-Wallis test was employed. The paired t-test or Wilcoxon signed rank test was used for comparing baseline values and the values at six months. For nominal data, the chi-square or Fisher’s exact test was employed. Spearman’s correlations were used to test for linear relationships between the variables measured on the interval scale. Unless specified otherwise, a two-tailed p-value of less than 0.05 was considered statistically significant. SAS® version 9.2 was used for all analyses.

The following modeling process was used to select variables among 27 immune analytes and 2 clinical lab test results (serum albumin or total bilirubin) for prediction of early death (within 6 month of DILI onset). Due to small sample size and the relatively large number of variables, the goal was to find a stable model (34) with small numbers of variables that are highly predictive of early death. In the first step, univariate analyses were carried out to compare those died within 6 months of DILI onset *vs.* those who survived by using the Wilcoxon rank sum test. To be highly selective, only those variables that were statistically significant at p < 0.01 level were considered in the second step. It was expected that the immune analytes within each of the following three groups are likely to be correlated: cytokines, chemokines and growth factors. Thus in the second step, we examined the pairwise correlations within each group. We started out with the immune analyte that had the highest association with early death (lowest p-value). Only immune analytes within the group that were not significantly correlated with this immune analyte were retained for further consideration. If the pairwise correlations of the remaining immune analytes were significant, then we excluded the immune analyte with the lower association with early death from the pair. In the third step, we excluded immune analytes within the group that were poorly modulated based on heat-maps profiles of expressions. As a final step, we obtained the final set of the variables by examining the pairwise correlations among the remaining immune analytes and the clinical lab values. If the pairwise correlations were significant, then we excluded the variable with the lower association with early death from the pair. 

 Once the final set of variables had been selected, the area under curve (AUC) was estimated for each variable to evaluate its potential prognostic and diagnostic value. Logistic regression model with all of these variables was fit to estimate the AUC based on the fitted model with linear combination of the variables as a predictor. The linear combination of these variables may not be the best predictor for early death because the values of the immune analytes can tend to be skewed. Thus, the following binary variables were created as predictors. The immune analytes were dichotomized at observed median values and the clinical lab values were dichotomized based on established clinical cut points. Two summary binary variables were created, one based on binary immune analytes only and the other based on both the binary immune analytes and the clinical lab data. We expect that the binary variable based on both immune analytes and clinical lab data to have the highest predictability for early death. This binary variable has a value of 1 if values of the variables (immune analytes and clinical lab values) all fall in the binary category that was predictive of early death based on direction of association, and a value of 0 otherwise. The predictability of this binary variable for early death was evaluated by positive predictive value (PPV), negative predictive value (NPV) and accuracy (percent of correct prediction). Sensitivity and specificity were also calculated.

## Results

### Characteristics of Study Cohorts

**Figure 2:**
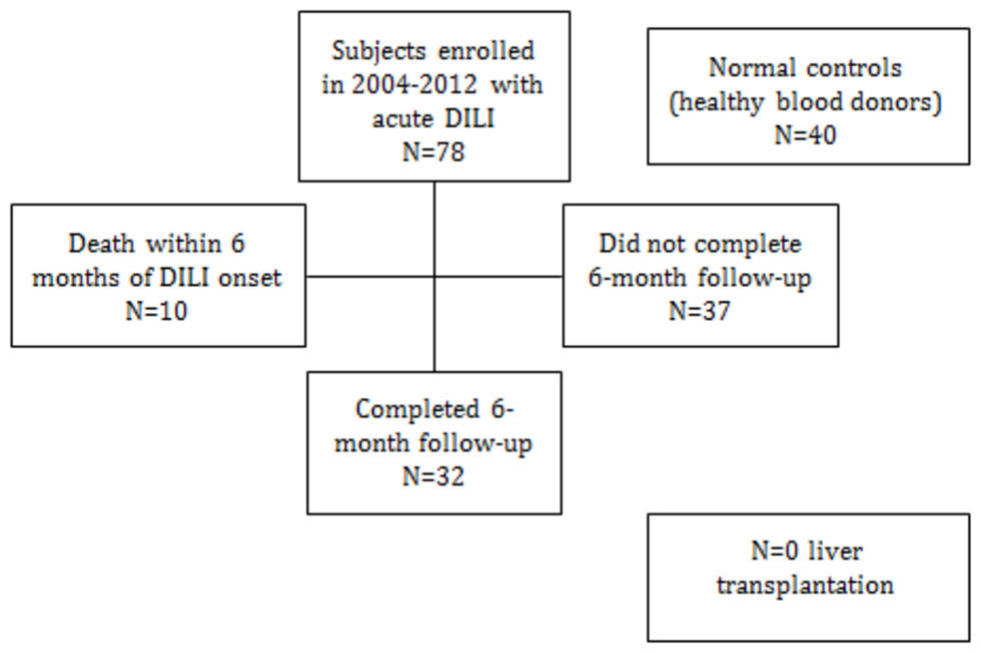
Flow diagram of study cohort. Sera from 78 DILI subjects and 40 healthy controls (volunteer blood donors) were analyzed in the study. Among those patients with acute DILI, 10 died within 6 months of DILI onset and 37 returned for a 6-month follow-up visit, among whom serum samples were obtained from 32.

Seventy-eight subjects with acute DILI and 40 healthy controls were included in the study [24] (Figure **2**). Thirty-seven subjects with DILI completed 6-month follow-up visits among whom, sera were obtained in 32 subjects. [Due to various reasons, 5 subjects did not provide serum samples at 6 months]. Ten subjects (12.8%) died within 6 months of DILI onset, whereas one died of a non-DILI cause after 315 days. None underwent liver transplantation. 

Demographic and lab data are presented in Table **1**. The mean age of the DILI cohort was 48 (±17.9) years old. Gender distribution was 55% women, and 73% were Caucasian [European-American]. As shown in Table **2**, 59%, 22%, 15% and 4% of DILI subjects, respectively, presented with hepatocellular, cholestatic, mixed or unknown patterns of liver injury, similar to those of the entire DILI cohort [8].

**Table 1:** Selected demographic, clinical, and laboratory features of subjects studied.

	**DILI onset (n=78)**	**6-month follow-up (n=32)**	**Healthy controls (n=40)**
**Age, mean ± SD (y**)	48 ± 17.9	51 ± 14.2	49.2 ± 13.1
**Female (%**)	55	55	28
**Self-reported race (%**)			
**White**	73	73	95
**Black**	10	10	5
**Other**	16	15	0
**Unknown**	1	0	0
**Body mass index, mean ± SD (kg/m^2^)**	27.1 ± 6.5		30.5 ± 6.9
**Absolute eosinophils/µL (mean ± SD)**	173± 235		
**Liver biochemistries, mean ± SD**			
**ALT** (**U/L**)	1065 ± 1382	55±131	17± 5
**AST** (**U/L**)	1003 ± 1249	38±47	24 ± 5
**Alkaline phosphatase** (**U/L**)	336± 465	89±33	63± 14
**Total bilirubin** (**mg/dL**)	8.1 ± 7.4	0.9±0.7	0.6 ± 0.2
**INR**	1.8 ± 1.2	1.1±0.4	

Abbreviations: DILI, Drug-induced liver injury; SD, standard deviation; ALT, alanine aminotransferase; AST, aspartate aminotransferase; INR, international normalized ratio.

**Table 2:** Selected DILI characteristics and outcomes among subjects studied [n= 78].

**Pattern of liver injury (%**)	
**Hepatocellular**	59
**Cholestatic**	22
**Mixed**	15
**Unknown**	4
**Severity of liver injury (%**)	
**Mild**	11
**Moderate**	49
**Severe/fatal**	26
**Unknown**	14
**Liver-related mortality (%**)	6
**Chronic DILI (%**)	5

### Longitudinal analysis: immune analytes and the course of DILI

Experimental models of DILI and retrospective clinical studies [12-15,17-21] have posited immune activation mechanisms associated with acute liver injury. In order to characterize immune components associated with DILI regardless of the pattern and severity of injuries, serum levels of immune analytes were compared among DILI onset (n=78), 6-month follow-up (n=32) and healthy controls (n=40). The three groups displayed a high degree of heterogeneity. For 26 of the 27 immune analytes, the results were not normally distributed. 

**Figure 3:**
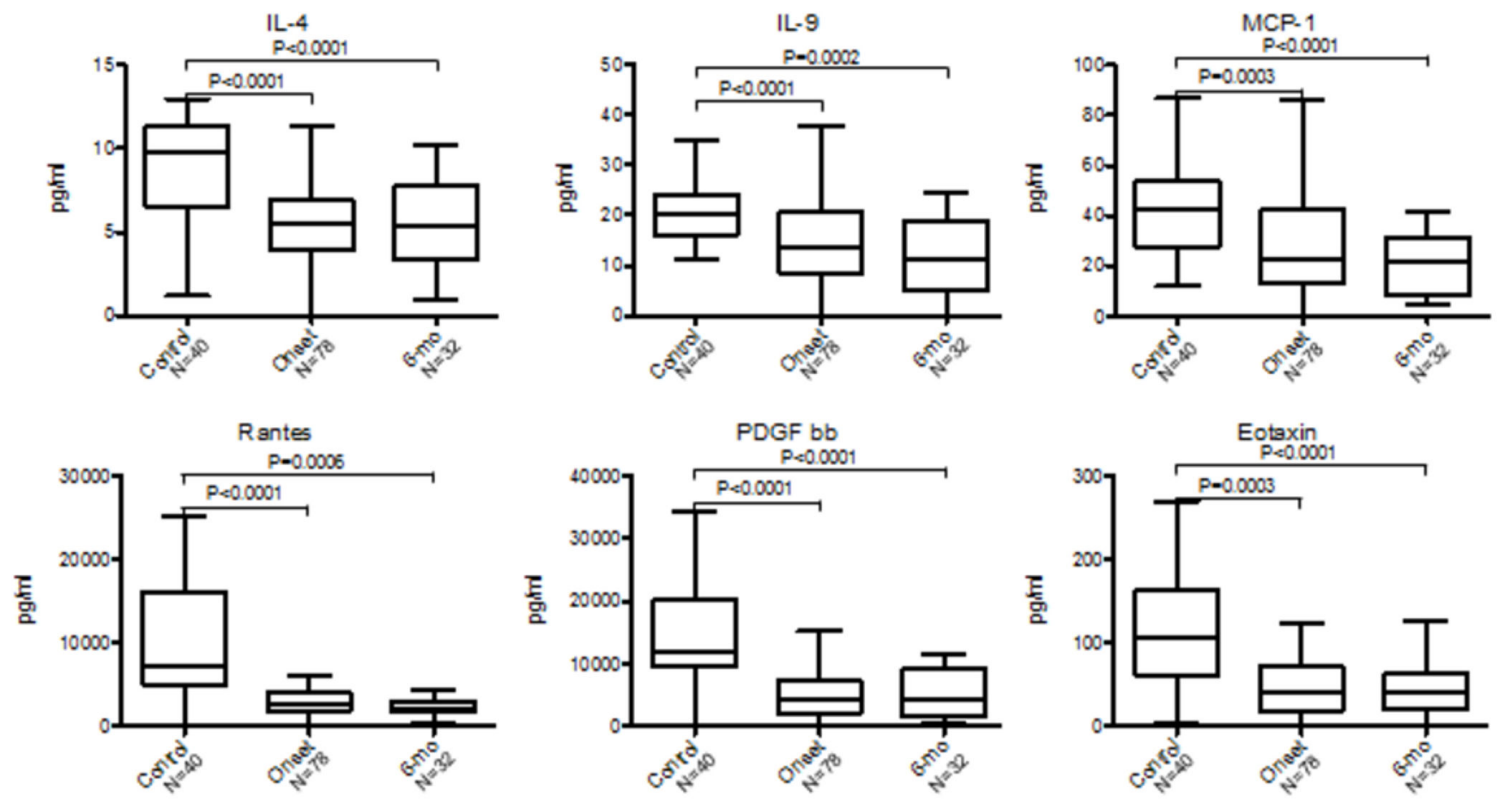
Immune analytes concentrations differentially distributed among control, DILI onset and 6-month follow up samples. Box-and whisker plot: Turkey representation (i.e. 25-75 percentile +/- 1.5 interquartile distance) of immune analytes concentration in sera collected from control, DILI onset and 6-month follow up. Immune analytes measurements are in pg/mL. * Comparisons of all distributions to control samples are statistically significant (p<0.05) by Wilcoxon rank sum test. § Comparisons of the difference between DILI onset and 6-month follow up distributions are statistically significant (p<0.05) by paired t-test or Wilcoxon rank sum test.

**Figure 4:**
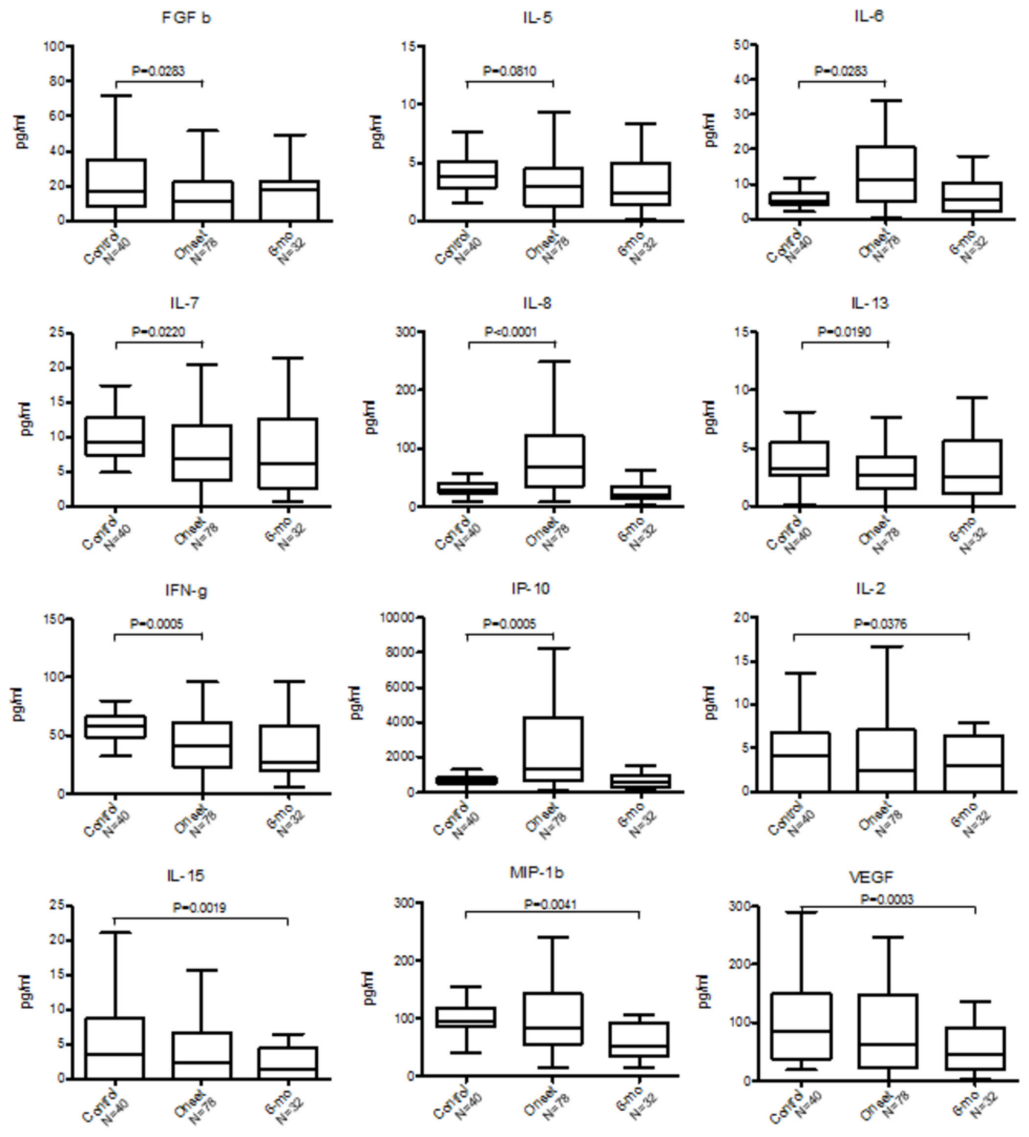
Immune analytes concentrations differentially distributed between control, DILI onset or 6-month follow up samples. Box-and whisker plot: Turkey representation (i.e. 25-75 percentile +/- 1.5 interquartile distance) of immune analytes concentration in sera collected from control, DILI onset and 6-month follow up. Immune analytes measurements are in pg/mL. * Comparisons of DILI onset or 6-month follow up distribution to control samples are statistically significant (p<0.05) by Wilcoxon rank sum test. § Comparisons of the difference between DILI onset and 6-month follow up distributions are statistically significant (p<0.05) by paired t-test or Wilcoxon rank sum test.

A Wilcoxon rank-sum test was performed to assess which cytokines, chemokines or growth factors, were altered in DILI (Figure **3** **, 4 and Table S1 **in File **S1**). Distributions of serum levels of 6 immune analytes significantly differed among healthy controls (n=40), DILI at onset (n=78) and DILI at 6-month follow up (n=32): IL-4, IL-9, eotaxin, RANTES, PDGF-bb, and MCP-1 (Figure **3**). In addition, serum levels of 8 immune analytes were differently distributed between control and DILI at onset (IL-5, IL-6, IL-7, IL-8,IL-13, IP-10, FGF basic, IFN-γ,), and 4 (IL-2, IL-15, VEGF, MIP-1β) were differently distributed between control and DILI at 6-month follow-up (Figure **4**). For those subjects with 6-month follow-up samples (n=32), the differences between the baseline and follow-up samples were examined. Significant changes were observed for 8 immune analytes (IL-5, IL-8, IP-10, IFNγ, FGF basic, MIP-1β, GM-CSF and VEGF) (**Table S2 **in File **S1**). Overall, among the 27 immune analytes investigated, 19 showed significant differences among healthy controls and DILI at onset and/or at 6-month follow-up. 

Among those who survived for more than 6 months, all showed marked clinical and biochemical improvement in DILI.

### Comparative analysis of immune analyte pattern(s) of DILI subjects

To provide a framework better to understand the heterogeneity observed in control, DILI onset, and DILI 6-month groups, immune profiles of individual DILI subjects were assessed and compared. These immune profiles, were established based on observed patterns of cytokine serum levels and from consideration of known immunological principles. While the function and origin of individual immune analytes can be determined, cytokines actually function in networks (synergy, antagonism). For instance, in a simplified model of immune processes (Figure **1**), an immune stimulus (e.g., cell damage caused by a drug or other chemical) triggers NFκB nuclear translocation and early/innate cytokine production (IL-1β, IL-6, TNF-α) by damaged tissue. If this early inflammatory state persists, it will activate adaptive immune processes favoring either cellular (T-bet-dependent / TH1-type: IL-12p70, IFNγ, IL-2, IL-15; RoRγt-dependent / TH17-type: IL-17), or humoral (Gata3-dependent / TH2-type: IL-4, IL-5, IL-13; IRF4-dependent / TH9-type: IL-9) responses [26,27,30]. If the inciting agent (in this case a causative drug) is removed, usually, immune responses will ultimately resolve themselves by triggering anti-inflammatory processes (IL-10, IL-1ra) [29].

**Figure 5:**
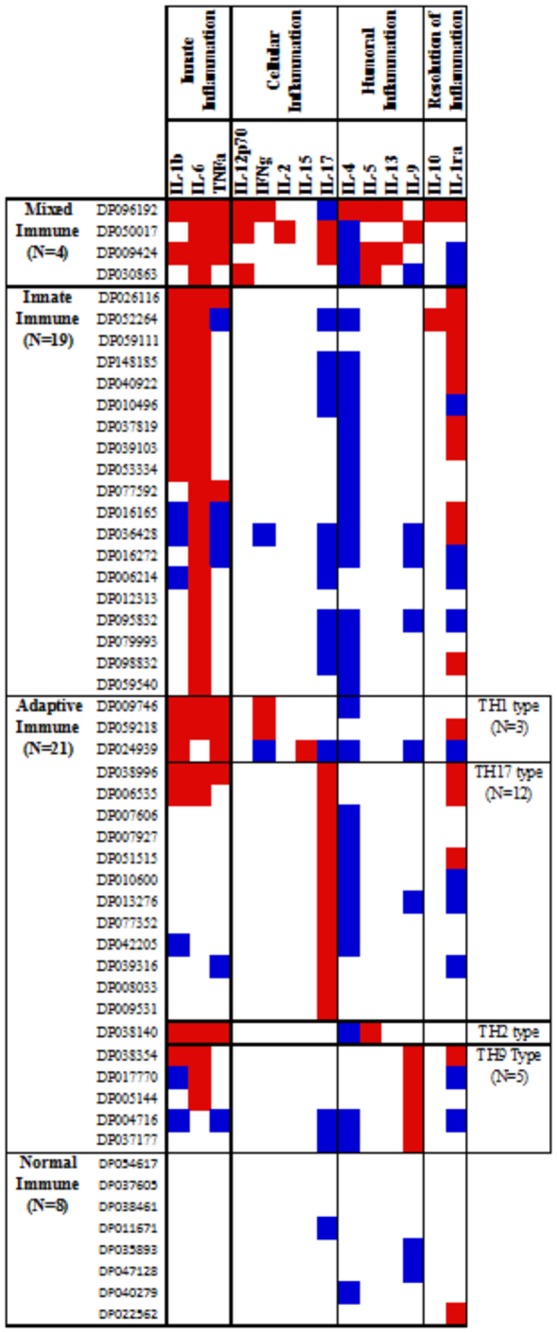
Profiles of immune analytes in serum at onset of DILI. Individual cytokine concentrations in sera obtained close to (within 14 days of) DILI onset were recorded for each patient and compared with healthy “normal” means ± SD values. Abnormal serum cytokine concentrations at DILI onset were defined as values that were higher (red) or lower (blue) than those of the means for normal controls. The profiles of DILI subjects at onset were defined based on observed similarities of patterns and upon knowledge of the physiologic roles of the analytes (see text and Fig 1).

Four distinct immune profiles emerged among DILI subjects at the onset of the disease (n=78) (Figure **5**, Figure **S1**). Nineteen subjects displayed an “innate immune” profile with one or more early/innate cytokines higher than normal. Twenty-one DILI subjects showed an “adaptive immune” profile with at least one adaptive cytokine higher than normal. Four DILI subjects had a “mixed-immune” profile (both early and innate cytokines higher than normal), and 8 a “normal-immune” profile (no abnormal innate and 1 or fewer abnormal adaptive cytokine levels). Twenty-six DILI subjects were uncategorized (2 or more abnormal cytokines levels). Of note, among the 21 DILI subjects with an “adaptive immune” cytokine profile, classical TH1 and TH2 adaptive responses were rare. Rather at DILI onset, TH17 (15.4%) and TH9 (6.4%) increases predominated (Figure **5**, Figure **S1**).

**Figure 6:**
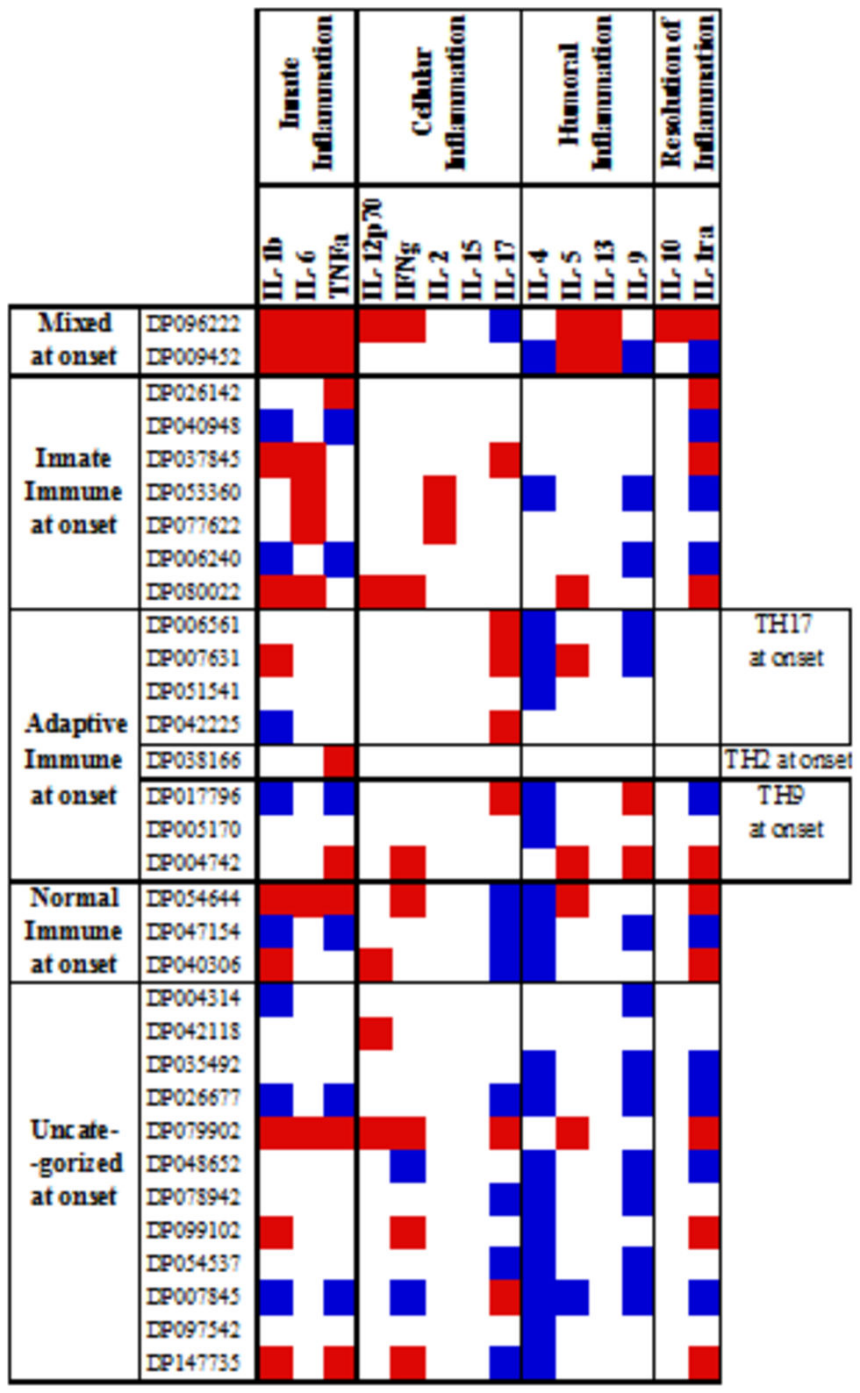
Profiles of cytokines in sera of DILI subjects at 6-month follow-up. Individual cytokine concentrations in 6-month follow-up sera were recorded for each patient and compared with healthy “normal” means ± SD values. Abnormal serum cytokine concentrations at 6-mo follow-up were defined as measurements SD higher (red) or lower (blue) than those of the means for normal controls. The profiles of DILI subjects at 6 month follow up were defined based on observed similarities of patterns and upon knowledge of the physiologic roles of the analytes (see text and Fig 1).

**Figure 7:**
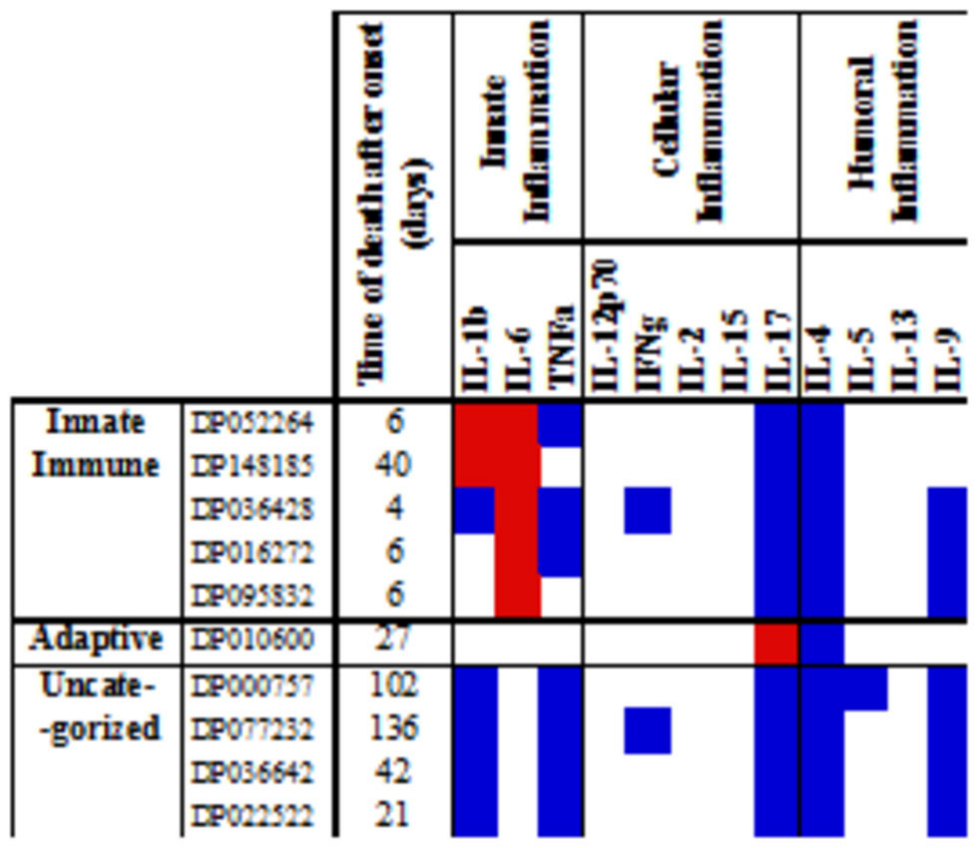
Cytokine profiles in sera of subjects who died within 6 months of DILI onset. Individual cytokine concentrations were recorded for each patient and compared with healthy “normal” means ± SD values. Abnormal serum cytokine concentrations were defined as measurements SD higher (red) or lower (blue) than those of the means for normal controls. The profiles of DILI subjects who died within 6 months of DILI onset were based on observed similarities of patterns and upon knowledge of the physiologic roles of the analytes (see text and Fig 1).

Using a similar methodology to compare the 32 DILI subjects for whom 6-month follow-up samples and data were available, at 6 months 12 exhibited normal or lower than normal serum cytokine levels and 17 DILI subjects showed higher than normal serum levels of adaptive cytokines (Figure **6**, Figure **S2**). Among the cohort of 32, over-expressed adaptive cytokines were predominantly TH1 (6/32) or cellular/humoral hybrid (6/32). Only a few displayed TH17 (4/32) or TH9 profiles (1/32) (Figure **7**, Figure **S3**).

### Immune Analytes and Clinical Features of DILI

Correlations among clinical features and cytokine levels were explored. Several cytokines, chemokines and growth factors were found to be significantly associated with hyperbilirubinemia (serum total bilirubin >1.2mg/dL) and self-reported jaundice (Table **3**). Those with hyperbilirubinemia or self-reported jaundice had mean serum ALT and AST levels that were not significantly different from those of other DILI subjects. The median values for nearly all of the immune analytes were higher for subjects with hyperbilirubinemia or self-reported jaundice compared to those without. No significant association was evident for the following clinical features and any of the cytokines measured: gender, age, pattern of DILI, severity of DILI, serum ALT at onset, peak serum ALT, serum AP at onset, peak serum AP, R value, BMI, absolute eosinophil count. There were also no significant associations among concentrations of immune analytes and individual drugs implicated in causing DILI (Table **4**). Grouping causative drugs into major therapeutic classes [e.g., anti-infectious agents, anti-convulsants, anti-hypertensive agents, lipid lowering agents, etc] did not uncover significant correlations among classes of drugs or their indications for use and immune profiles. Rather, causative agents were to be found in several different types of immune response.

**Table 3:** Serum levels at DILI onset of immune analytes significantly associated with an elevation of serum total bilirubin.

	**Serum Total Bilirubin >1.2 mg/dL (n = 59)**			**Serum Total Bilirubin 0 - 1.2 mg/dL (n = 17)**			p value^*^
	Median	Min.	Max.	Median	Min.	Max.	
**IL-1β**	1.0	0.0	48.2	0.6	0.0	2.5	0.035
**IL-1ra**	145.6	29.1	719.6	81.6	4.7	1406	0.007
**IL-5**	3.1	0.5	68.4	2.1	0.0	6.5	0.0156
**IL-7**	8.0	1.4	165.0	3.7	0.0	13.8	0.0131
**IL-8**	82.7	15.6	4636	42.7	7.4	463.1	0.0043
**IP-10**	2163.6	222.6	48050	835.2	71.9	39266	0.0124
**MIP-1α**	3.6	0.0	80.1	1.4	0.0	12.0	0.0184
**MIP-1β**	99.5	19.3	473.8	54.7	15.6	158.4	0.0011
**PDGF-bb**	4819	65.3	28757	1772.5	130.9	15951	0.0101

**All results are pg/mL**. Values listed as zero are out of assay operating range.

^*^ Comparisons of distributions are statistically significant (p<0.05) by Wilcoxon rank-sum test.

**Table 4:** Drugs implicated in causing DILI sorted by immune profiles at DILI onset.

		**Implicated agent 1**	**Implicated agent 2**	**Implicated agent 3**				**Implicated agent 1**	**Implicated agent 2**	**Implicated agent 3**
**Mixed-immune**	DP096192	Simvastatin^2^				**Normal-immune**	DP054617	Minocycline^1^		
	DP050017	Pregabalin^3^					DP037605	Drospirenone & Ethynylestradiol^5^		
	DP009424	Sertraline^3^					DP038461	Methyldopa^4^		
	DP030863	Orlistat^2^	Sulfametho-xazole & Trimethoprim^1^				DP011671	Atorvastatin^1^		
**Innate immune**	DP026116	Levofloxacin^1^					DP035893	Isoniazid^1^		
	DP052264	Antithymocyte globulin^5^					DP047128	Minocycline^1^		
	DP059111	Phenytoin^3^					DP040279	Sulfametho-xazole & Trimethoprim^1^		
	DP148185	Amiodarone^4^					DP022562	Metformin^2^	Fenofibrate^2^	
	DP040922	Herbal preparation^5^				**Uncate-gorized**	DP000757	Oxacillin^1^		
	DP010496	Isoniazid^1^	Rifampicin^1^				DP009853	Amoxicillin & Clavulanic acid^1^		
	DP037819	Phenylpropano—lamine^5^					DP077232	Vincristine^2^	Asparaginase^5^	
	DP039103	Ribabutin^1^					DP004288	Moxifloxacin^1^	Ciprofloxacin^1^	Amoxicillin & Clavulanic acid^1^
	DP053334	Isoniazid^1^					DP037073	Amitryptyline^3^	Nicotinic Acid^2^	
	DP077592	Flavocoxid^5^	Pregabalin^3^				DP036642	Amlodipine^4^		
	DP016165	Octreotide^2^					DP022522	Carbamazepine^3^	Sulfametho-xazole & Trimethoprim^1^	Lisinopril^4^
	DP036428	Investigational drug^5^					DP049910	Herbal preparation^5^	Panax ginseng^5^	Ginkgo biloba^5^
	DP016272	Nitrofurantoin^1^					DP042092	Nicotinic Acid^2^		
	DP006214	Isoniazid^1^					DP022492	Allopurinol^2^	Fluconazole^1^	Cyclophos-phamide^5^
	DP012313	Lamotrigine^3^					DP038889	Rosuvastatin^2^		
	DP095832	Nicotinic Acid^2^					DP035467	Duloxetine^3^		
	DP079993	Valproic acid^3^	Amiodarone^4^	Amoxicillin & Clavulanic acid^1^			DP009211	Methyldopa^4^		
	DP098832	Lamotrigine^3^	Ziprasidone^3^				DP026651	Disulfiram^5^	Lisinopril^4^	
	DP059540	Azathioprine^5^					DP079872	Herbal preparation^5^		
**Adaptive immune**	DP009746	Amoxicillin & Clavulanic acid^1^			**TH1 type**		DP048626	Dicloxacillin^1^	Amoxicillin & Clavulanic acid^1^	
	DP059218	Metoprolol^4^					DP078913	Atripla^1^ [efavirenz, emtricitabine, tenofovir]		
	DP024939	Antithymocyte globulin^5^					DP099072	Azithromycin^1^		
	DP038996	Fenofibrate^2^			**TH17 type**		DP008461	Telithromycin^1^		
	DP006535	Isoflurane^5^	Lorazepam^3^	Clindamycin^1^			DP054511	Amoxicillin & Clavulanic acid^1^		
	DP007606	Linezolid^1^					DP026334	Lamotrigine^3^	Valproic acid^3^	
	DP007927	Tetracycline^1^	Sulfametho-xazole & Trimethoprim^1^				DP007819	Nitrofurantoin^1^		
	DP051515	Chloroxazone^5^					DP097512	Anabolic steroids^5^		
	DP010600	Ciprofloxacin^1^	Metronidazole^1^				DP078432	Herbal preparation^5^		
	DP013276	Nitrofurantoin^1^					DP041996	Methyldopa^4^	Labetalol^4^	
	DP077352	Ceftriaxone^1^	Ampicillin^1^	Fluconazole^1^			DP147705	Voriconazole^1^	Fluconazole^1^	
	DP042205	Isoniazid^1^								
	DP039316	Isoniazid^1^								
	DP008033	Phenytoin^3^	Levofloxacin^1^	Phenobarbital^3^						
	DP009531	Sulfametho-xazole & Trimethoprim^1^								
	DP038140	Valaciclovir^1^			**TH2 type**					
	DP038354	Herbal preparation^5^			**TH9 type**					
	DP017770	Bortezomib^5^								
	DP005144	Darunavir^1^	Didanosine^1^	Abacavir & Lamivudine^1^						
	DP004716	Pregabalin^3^	Simvastatin^2^	Mercaptopurine^5^						
	DP037177	Allopurinol^5^	Rosiglitazone^2^							

^1^Anti-infectious agents; ^2^Lipid lowering and anti-diabetic agents; ^3^ Anti-depressant and ant-convulsant agents; ^4^ Anti-hypertensive agents / cardiac; ^5^ other

Among the 10 subjects who died within 6 months following DILI onset, 5 displayed an “innate DILI” cytokine profile while only 1 had a TH17 signature (Figure **7**, Figure **S3**). The remaining 4 subjects had an “uncategorized” cytokine profile, and deaths tended to occur later in these subjects compared with those with “innate DILI” profile (median time of death following DILI onset 72 days vs. 6 days).

### Immune Analytes and DILI Outcomes

Our modeling process selected low levels of 4 immune analytes (IL-9, IL-17, PDGF-bb and RANTES) and serum albumin to be used for prediction. Step 1 of the modeling process showed that 7 cytokines (TNFα, IL-12, IL-17, IL-4, IL-5, IL-13, IL-9), 2 chemokines (MIP-1β, RANTES), 2 growth factors (FGFb, PDGF-bb) and albumin were significantly (at level of 0.01) associated with death within 6 months of DILI onset based on univariate analyses (Table **5**). Four cytokines (TNFα, IL-4, IL-5, IL-12) and one growth factor (FGFb) were excluded in step 2 due to their significant correlations with the highest ranked cytokine IL-17 and highest ranked growth factor PDGF-bb (based on p-values). Cytokine IL-13 was excluded in step 3 because it was poorly modulated based on the heat map of cytokine expression profile (Figure **5**, Figure **S1**). MIP-1β was excluded in the final step due to its significant correlation with growth factor PDGF-bb.

**Table 5:** Serum levels at DILI onset of immune analytes significantly associated with death within 6 months of DILI onset.

	**Died [n = 10]**			**Survived [n = 68]**			p value^*^
	Median	Min.	Max.	Median	Min.	Max.	
**Cytokines**							
**TNFα**	0.5	0.0	30.9	20.1	0.0	326.5	0.001
**IL-12**	3.1	0.0	27.4	16.7	0.0	2185.6	<0.001
**IL-17**	8.6	0.0	156.7	47.4	0.0	139.1	<0.001
**IL-4**	2.3	0.0	5.8	5.8	0.5	12.7	<0.001
**IL-5**	1.1	0.0	4.9	3.0	0.0	68.4	0.006
**IL-13**	0.7	0.0	4.0	2.8	0.0	130.1	<0.001
**IL-9**	6.7	0.0	17.1	15.5	0.0	2194.2	0.003
**Chemokines**							
**MIP-1β**	55.1	19.3	133.1	92.4	15.6	473.8	0.006
**RANTES**	1412.8	190.0	2301.2	2740.6.8	761.4	65180	<0.001
**Growth Factors**							
**FGFb**	0.0	0.0	15.7	13.5	0.0	67.9	0.003
**PDGF-bb**	487.0	65.3	7313.1	4719.4	130.9	28757	<0.001
**Clinical Lab**							
**Albumin**	2.3	1.8	2.8	3.1	1.8	4.9	0.001

**Analyte results are pg/mL**. Values listed as zero are out of assay operating range.

^*^ Comparisons of distributions are statistically significant (p≤0.01) by Wilcoxon rank sum test

 Low values of immune analytes and low values of serum albumin were predictive of early death with estimated AUCs (95% CI) of 0.79 (0.65, 0.93) for IL-9, 0.86 (0.67, 1.0) for IL-17, 0.84 (0.68, 1.0) for PDGF-bb, 0.90 (0.81-0.98) for RANTES, and 0.83 (0.73, 0.94) for serum albumin. The AUC (95% CI) of the linear combination of these variables based on the logistic regression model was 0.98 (0.95, 1.0). Because the values of the immune analytes were highly skewed, binary variables were created to combine and to summarize the information from these variables. The observed median values of the four immune analytes and the known cut point of 2.8 g/dL (used in the Child-Turcotte-Pugh scoring system for identifying subjects with severe [class C] liver disease) for albumin were used to dichotomize the continuous variables into binary variables. Subjects in the DILI cohort with a baseline value of serum albumin greater than 2.8 g/dL all survived with 100% NPV, whereas all of those who died within 6 months of DILI onset had values below this cutoff with 100% sensitivity (Table **6**). The binary variable based on the final four immune analytes (IL-9, IL-17, PDGF-bb and RANTES) had estimated PPV, NPV and accuracy of 67%, 97% and 92%, respectively, if values of four immune analytes were all below the observed medians. Combination of the four binary immune analytes and binary serum albumin provided optimal differentiation between those who died of acute DILI or survived for at least six months (and usually recovered completely). Using this benchmark, 67 (97% NPV) out of 69 subjects with IL-9 >13.6, IL-17 >45.2, PDGF-bb >4207, RANTES >2529 (all pg/mL) or albumin >2.8g/dL survived the acute DILI event at 6 months, whereas 7 (88% PPV) out of 8 subjects with IL-9 <13.6, IL-17 <45.2, PDGF-bb <4207, RANTES <2529 (all pg/mL) and albumin ≤2.8g/dL died within 6 months of DILI onset (Table **6**). The overall accuracy of prediction is 74/77=96% (95% CI, 92%-100%). Baseline MELD scores were also explored for predictability for early death. As shown in Table **7**, MELD scores [31] also were predictive , albeit not as accurate as the combined binary variable of 4 immune analytes and albumin. 

**Table 6:** Binary Variables predictive of death within 6 months of DILI onset.

	**Acute death (< 6 months)**	**SurvivalAt 6-months**	**Sensitivity (95% CI)**	**Specificity (95% CI)**	**Positive predictive value (95% CI)**	**Negative predictive value (95% CI)**	**Accuracy (95% CI)**
Serum albumin (N=74)*							
**≤ 2.8g/dL**	9	26	100% (NA)	60% (48%, 72%)	26% (11%, 40%)	100% (NA)	65% (54%, 76%)
**> 2.8g/dL**	0	39					
**Four immune analytes (IL-9, IL_17, PDGF-bb RANTES) (N=78)**							
**All immune analytes below medians** **	8	4	80% (55%, 100%)	94% (89%, 100%)	67% (40%, 67%)	97% (93%, 100%)	92% (86%, 98%)
**At least one above median**	2	64					
Four immune analytes (IL-9, IL_17, PDGF-bb RANTES) and serum albumin (N=77)***							
All immune analytes below medians** and albumin ≤ 2.8 g/dL	7	1	78% (51%, 100%)	99% (96%, 100%)	88% (65%, 100%)	97% (93%, 100%)	96% (92%, 100%)
**At least one immune analyte above median or albumin > 2.8 g/dL**	2	67					

^*^ Four subjects were excluded due to missing values of serum albumin at DILI onset

^**^ IL-9 <13.6, IL-17 <45.2, PDGF-bb<4207, RANTES<2529 (all pg/ml) where observed medians for 78 subjects.

^***^ One subject was excluded whose category could not be determined due to missing serum albumin result and values of all four immune analytes were below the medians.

**Table 7:** Deaths actual vs. predicted, using MELD scores at baseline for DILI subjects (n=34).

MELD Score	n	Actual Deaths	Predicted Deaths
**>40**	2	0/2 = 0%	71.3%
**30-39**	5	4/5 = 80%	52.6%
**20-29**	10	2/10 = 20%	19.6%
**10-19**	16	0/16 = 0%	6.0%
**<10**	1	0/1 = 0%	1.9%

[Unfortunately, all data necessary for the calculation of MELD scores were available for only 34 of the 78 subjects studied, including 6 of the 10 who died within six months. The values often missing were the serum creatinine and INR.]

## Discussion

We performed extensive profiling of serum immune analytes in subjects with carefully studied, prospectively followed acute DILI, and compared results to those of normal controls. The main findings of this work are that 1) profiles of serum immune analytes are altered in acute DILI; 2) such profiles may be classified into different types; 3) profiles are not specific to or correlated with the underlying drug causes of DILI, with the pattern of DILI, whether hepatocellular, cholestatic, or “mixed”, nor with the R value at baseline or at later time points; and 4) low values of serum albumin and a summary variable of only four immune analytes at DILI onset (IL-17, IL-9, PDGF-bb and RANTES) are predictive of who will survive and who will not survive for 6 months after an acute DILI event. These results, if confirmed, will advance our fundamental understanding of the pathogenesis and progression of DILI and our ability to predict early in the course of acute DILI those subjects who are likely to survive or not survive.

With respect to advancing our understanding of pathogenesis of DILI, differing immune response patterns are suggested from analysis of cytokine profiling of DILI subjects that we believe provide valuable new insights. It is increasingly clear that DILI is often due to an exuberant immunological response to a drug (or drug metabolite), usually bound to host proteins. For example, serum albumin has been implicated as a frequent carrier of drug-derived haptens [32,33]. These complexes have been linked to immune responses that lead to liver injury [10,34]. Such responses, directed chiefly at hepatocytes or at bile ductular cells, may also attack other organs and tissues, particularly the skin, the kidneys or the bone marrow [35]. The current paradigms for immunological characterization of DILI [e.g., as auto-immune-like or immuno-allergic] are based on clinico-pathological manifestations of the disease, presence of auto-antibodies (adaptive immunity), eosinophilia (innate immunity) and/or skin rash [10,36]. Auto-immune type DILI tends to have a delayed onset and tends to evolve slowly on re-challenge [37]. Skin rashes, a mark of immuno-allergic-like DILI, can be triggered by either innate immune or adaptive immune components (e.g., DRESS syndrome, systemic lupus erythematosus) [38,39]. Taken together, the prototypical auto-immune-like or immuno-allergic categories of DILI show overlap, which is in keeping with clinical experience, with data in the US DILIN database, and with recent clinical reports from DILIN [[40-43], Rockey et al, Russo et al, unpublished observations]. Our cytokine profiling analyses highlight the innate immune component in DILI (19/78 subjects), and we found a distinct adaptive immune component at DILI onset that is mostly cellular (21/78 subjects). Interestingly, abnormal classical or canonical TH1 and TH2 adaptive cytokine expressions were rare in the DILI cohort investigated (4/78 subjects), whereas exuberant TH17 and TH9 adaptive immune responses were more prevalent (17/78 subjects). 

 This study has several strengths: we have carefully studied 78 subjects with DILI of known acute onset. We have extensive historical and clinical data on these subjects, and we have cytokine profiles performed with state-of-the-art methods. We have developed a novel modeling process for data reduction utilizing immune principles. The final single binary predictor is relatively easy to use, requiring measurement of only serum albumin and four immune analytes early in the course of the disease. This binary predictor seems to have high accuracy in predicting death of acute DILI [Table **6**]. Of particular importance is that two fundamentally different strategies employed to analyze immune analyte measurements, i.e., a strategy based upon the known biological function and regulation of immune analytes and a statistical/mathematical strategy, based upon the results observed in these studies, converged to give two main findings: 1) IL-17 (and to a lesser extent IL-9) are key contributors to the pathology of DILI at onset (more precisely DILI with an “adaptive immune” profile), and 2) low IL-17/IL-9 serum concentrations are associated with poor prognosis of DILI, or reciprocally, DILI with an “adaptive immune” profile is associated with a good prognosis.

Our results also suggest that subjects with acute DILI who have low levels of IL-9, IL-17, PDGF bb, RANTES, and serum albumin are at especially high risk of dying. Thus, perhaps, they should be treated with glucocorticoids or other immuno-modulating drugs and should be considered early for liver transplantation, because of their greater risk of acute death. They also might reasonably be the focus of possible new drugs or therapeutic approaches, such as the use of IL-10 or other anti-inflammatory biologic agents. Nevertheless, recent studies in patients with other forms of inflammatory liver diseases have not shown benefit of anti-TNF agents [44-47] or IL-10 [48-50], and the use of any of these agents would likely increase the risk of infections. Thus, they should be used in acute DILI only in the context of carefully designed clinical trials.

 A relative weakness of the study is related to the variation of individual cytokine measurements among subjects at DILI onset. Establishing cytokine profiles proved heuristically useful in that it allowed the identification of recurrent immune patterns and helped to order a large data set, but as many as a third of the patients did not fall into a unique, defined immune group. Such heterogeneity is not unexpected considering the nature of the cohort investigated (numerous causative drugs, various underlying conditions…). Another key component of immune profiling, namely, timing, also is worthy of comment. Each sample represents a snapshot in time, and cytokine profiles may be different at different time points. To minimize such possible effects, all subjects in our cohort were enrolled within 14 days of DILI onset. Because comparisons of the profiles for those enrolled within the first 7 days *vs.* those enrolled between days 7 and 14 after onset did not reveal any significant differences [data not shown], it is unlikely that enrolling subjects earlier [e.g., within 7 days] will reduce variability. Nonetheless, it would have been preferable to have had additional time points and samples, however, the design of the study did not include any. Thus, the heterogeneity of the subjects studied [particularly, the large number of inciting drugs, different genders, ages, ethnicity, etc] and the fact that, at baseline we were able to study only a single time point, are limitations of our study. Because of the relative rarity of acute DILI, we nevertheless believe that our data are the most extensive thus far obtained and analyzed carefully and, thus, represent useful and important new knowledge.

 In summary, there are characteristic profiles of cytokines in sera of subjects with acute, clinically important DILI, and these profiles appear to be useful for predicting who will live or will die or require liver transplant within six months of DILI onset. We plan to investigate whether these results will be confirmed in larger cohorts and to compare them to the profiles that develop in subjects with other types of acute liver injury. We hope that other investigative groups will do so as well.

## Supporting Information

Figure S1 **Profiles of immune analytes in serum at onset of DILI.** Individual cytokine concentrations in sera obtained close to (within 14 days of) DILI onset were recorded for each patient and compared with healthy “normal” means ± SD values. Abnormal serum cytokine concentrations at DILI onset were defined as values that were higher (red) or lower (blue) than those of the means for normal controls. The profiles of DILI subjects at onset were defined based on observed similarities of patterns and upon knowledge of the physiologic roles of the analytes (see text and Fig 1).
(TIF)

Figure S2 **Profiles of cytokines in sera of DILI subjects at 6-month follow-up.** Individual cytokine concentrations in 6-month follow-up sera were recorded for each patient and compared with healthy “normal” means ± SD values. Abnormal serum cytokine concentrations at 6-mo follow-up were defined as measurements SD higher (red) or lower (blue) than those of the means for normal controls. The profiles of DILI subjects at 6 month follow up were defined based on observed similarities of patterns and upon knowledge of the physiologic roles of the analytes (see text and Figure 1).
(TIF)

Figure S3 **Cytokine profiles in sera of subjects who died within 6 months of DILI onset.** Individual cytokine concentrations were recorded for each patient and compared with healthy “normal” means ± SD values. Abnormal serum cytokine concentrations were defined as measurements SD higher (red) or lower (blue) than those of the means for normal controls. The profiles of DILI subjects who died within 6 months of DILI onset were based on observed similarities of patterns and upon knowledge of the physiologic roles of the analytes (see text and Figure 1).
(TIF)

File S1 **Contains all supporting tables.**
(DOCX)
