# Supplementary figures and images for: Profiles of Serum Cytokines in Acute Drug-Induced Liver Injury and Their Prognostic Significance
Source: PLoS One. 2013 Dec 27;8(12):e81974. doi: 10.1371/journal.pone.0081974 (PMC3873930; doi:10.1371/journal.pone.0081974)

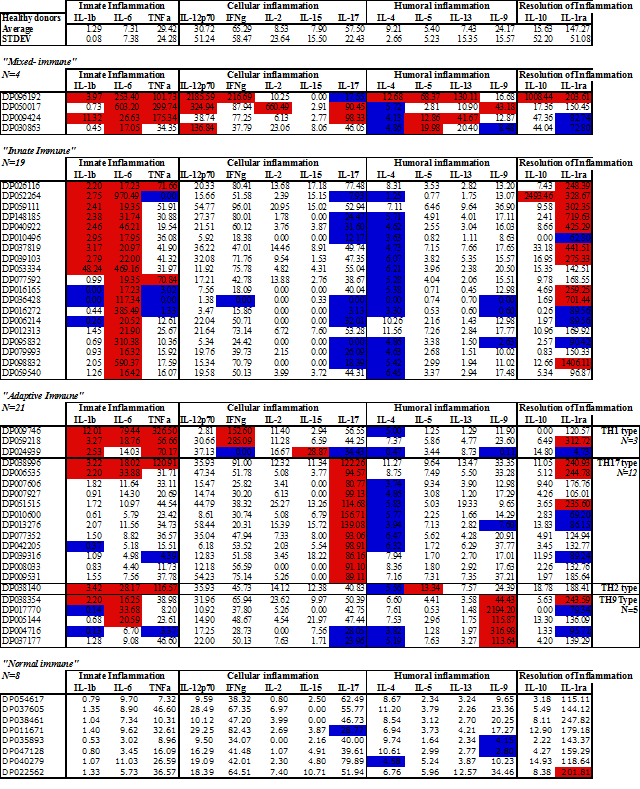

Supplement: Figure S1 — Profiles of immune analytes in serum at onset of DILI. Individual cytokine concentrations in sera obtained close to (within 14 days of) DILI onset were recorded for each patient and compared with healthy “normal” means ± SD values. Abnormal serum cytokine concentrations at DILI onset were defined as values that were higher (red) or lower (blue) than those of the means for normal controls. The profiles of DILI subjects at onset were defined based on observed similarities of patterns and upon knowledge of the physiologic roles of the analytes (see text and Fig 1). (TIF) [file pone.0081974.s001.tif]

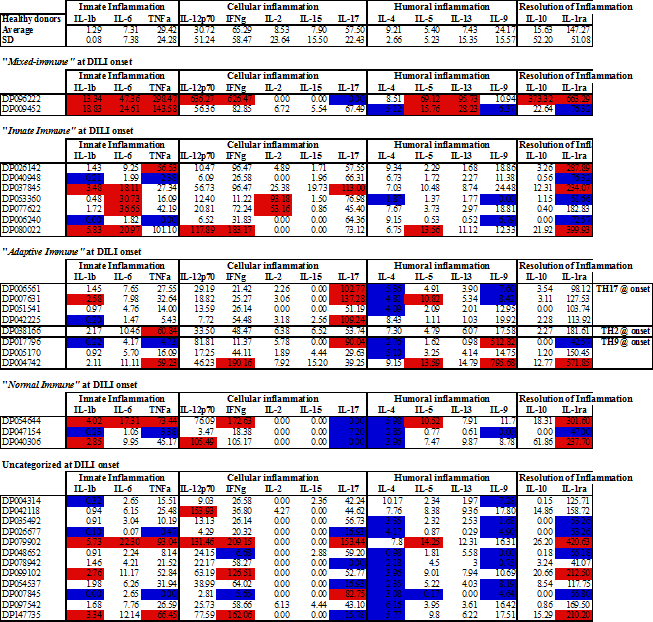

Supplement: Figure S2 — Profiles of cytokines in sera of DILI subjects at 6-month follow-up. Individual cytokine concentrations in 6-month follow-up sera were recorded for each patient and compared with healthy “normal” means ± SD values. Abnormal serum cytokine concentrations at 6-mo follow-up were defined as measurements SD higher (red) or lower (blue) than those of the means for normal controls. The profiles of DILI subjects at 6 month follow up were defined based on observed similarities of patterns and upon knowledge of the physiologic roles of the analytes (see text and Figure 1). (TIF) [file pone.0081974.s002.tif]

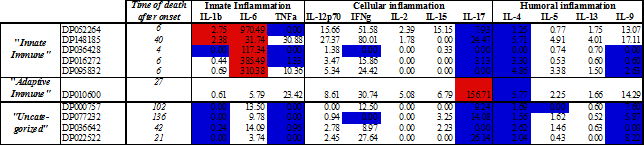

Supplement: Figure S3 — Cytokine profiles in sera of subjects who died within 6 months of DILI onset. Individual cytokine concentrations were recorded for each patient and compared with healthy “normal” means ± SD values. Abnormal serum cytokine concentrations were defined as measurements SD higher (red) or lower (blue) than those of the means for normal controls. The profiles of DILI subjects who died within 6 months of DILI onset were based on observed similarities of patterns and upon knowledge of the physiologic roles of the analytes (see text and Figure 1). (TIF) [file pone.0081974.s003.tif]
