# Supplementary material for: Profiles of Serum Cytokines in Acute Drug-Induced Liver Injury and Their Prognostic Significance
Source: PLoS One. 2013 Dec 27;8(12):e81974. doi: 10.1371/journal.pone.0081974 (PMC3873930; doi:10.1371/journal.pone.0081974)
Supplement: File S1 — Contains all supporting tables. (DOCX) [file pone.0081974.s004.docx]

**Table S1: Summary of immune analyte serum levels at DILI onset, 6-month follow-up, and in controls.**

|  | **DILI onset** | | | **6-month follow-up** | | | **Control** | | |
| --- | --- | --- | --- | --- | --- | --- | --- | --- | --- |
|  | Median | Min. | Max. | Median | Min. | Max. | Median | Min. | Max. |
| **Cytokines associated with** |  |  |  |  |  |  |  |  |  |
| **Innate Immunity** |  |  |  |  |  |  |  |  |  |
| **IL-1β** | 0.9 | 0.0 | 48.2 | 1.5 | 0.0 | 18.8 | 1.2 | 0.0 | 4.8 |
| **IL-6^b^** | 11.0 | 0.4 | 970.5 | 7.7 | 0.1 | 47.4 | 5.1 | 2.2 | 45.7 |
| **TNFα** | 17.1 | 0.0 | 326.5 | 27.0 | 0.0 | 298.5 | 22.7 | 0.0 | 116.6 |
| **Adaptive Cellular Immunity** |  |  |  |  |  |  |  |  |  |
| **IL-12** | 15.7 | 0.0 | 2186 | 24.9 | 2.8 | 636.3 | 16.0 | 5.7 | 294.8 |
| **IFNγ^b^** | 41.6 | 0.0 | 285.1 | 51.5 | 5.7 | 626.5 | 58.5 | 11.5 | 413.0 |
| **IL-2^c^** | 2.4 | 0.0 | 660.5 | 0.0 | 0.0 | 93.2 | 4.2 | 0.0 | 148.8 |
| **IL-15^c^** | 2.4 | 0.0 | 28.9 | 0.0 | 0.0 | 19.7 | 3.6 | 0.0 | 94.2 |
| **IL-17** | 45.2 | 0.0 | 156.7 | 53.3 | 0.0 | 153.4 | 52.7 | 23.5 | 118.3 |
| **Adaptive Humoral Immunity** |  |  |  |  |  |  |  |  |  |
| **IL-4^a^** | 5.6 | 0.0 | 12.7 | 5.6 | 1.0 | 10.2 | 9.8 | 1.2 | 12.9 |
| **IL-5^b^** | 3.0 | 0.0 | 68.4 | 4.2 | 0.2 | 69.1 | 3.8 | 1.5 | 27.6 |
| **IL-13^b^** | 2.7 | 0.0 | 130.1 | 4.0 | 0.0 | 95.7 | 3.24 | 0.2 | 95.0 |
| **IL-9^a^** | 13.6 | 0.0 | 2194.0 | 11.2 | 0.0 | 795.7 | 20.0 | 11.2 | 99.1 |
| **Immuno-suppression/ resolution** |  |  |  |  |  |  |  |  |  |
| **IL-1ra** | 137.7 | 4.7 | 1406 | 126.6 | 41.1 | 663.3 | 145.4 | 18.8 | 284.4 |
| **IL-10** | 4.3 | 0.0 | 2494 | 2.7 | 0.0 | 373.3 | 3.0 | 0.0 | 287.5 |
| **Chemokines** |  |  |  |  |  |  |  |  |  |
| **Eotaxin^a^** | 41.2 | 0.0 | 195.3 | 44.8 | 0.0 | 365.7 | 106.4 | 3.3 | 808.0 |
| **IL-8^b^** | 69.1 | 7.4 | 4636.0 | 28.6 | 4.1 | 141.5 | 28.5 | 8.6 | 82.0 |
| **IP-10^b^** | 1378.5 | 71.9 | 48050 | 749.9 | 177.7 | 7337.5 | 625.8 | 20.1 | 1663 |
| **MCP-1^a^** | 22.64 | 0.0 | 399.3 | 23.8 | 0.0 | 66.2 | 42.4 | 12.1 | 147.5 |
| **MIP-1α** | 3.4 | 0.0 | 80.1 | 3.9 | 0.0 | 27.6 | 3.5 | 1.7 | 30.3 |
| **MIP-1β^c^** | 84.1 | 15.6 | 473.6 | 72.5 | 15.2 | 223.4 | 95.7 | 5.3 | 190.2 |
| **RANTES^a^** | 2529.0 | 190.0 | 65180 | 2615.2 | 441.4 | 43545 | 7227.4 | 14.5 | 65180 |
| **Growth Factors** |  |  |  |  |  |  |  |  |  |
| **IL-7^b^** | 7.0 | 0.0 | 165.0 | 10.4 | 0.7 | 103.2 | 9.4 | 5.0 | 104.1 |
| **FGF b^b^** | 10.9 | 0.0 | 67.9 | 21.5 | 0.0 | 164.5 | 16.6 | 0.0 | 108.2 |
| **G-CSF** | 19.0 | 5.7 | 425.1 | 17.8 | 2.7 | 118.7 | 21.7 | 9.3 | 61.4 |
| **GM-CSF** | 0.0 | 0.0 | 86.7 | 0.0 | 0.0 | 23.6 | 0.0 | 0.0 | 413.4 |
| **PDGF-bb^a^** | 4207.0 | 65.3 | 28757 | 3595.1 | 697.2 | 23350 | 12037 | 12.1 | 34353 |
| **VEGF^c^** | 62.4 | 0.0 | 620.8 | 42.3 | 3.1 | 136.6 | 84.4 | 19.0 | 411.6 |

**All results are pg/mL.** Values listed as zero are out of assay operating range.

**^a^** Comparisons of all distributions to control samples are statistically significant (p<0.05) by Wilcoxon rank sum test.

**^b^** Comparisons of the onset distributions to control samples are statistically significant (p<0.05) by Wilcoxon rank sum test.

**^c^** Comparisons of the 6-month follow-up distributions to control samples are statistically significant (p<0.05) by Wilcoxon rank sum test.

**Table S2: Summary of immune analyte serum levels at DILI onset, 6-month follow-up, for N = 32 subjects with 6-month data.**

|  | **DILI onset** | | | **6-month follow-up** | | |
| --- | --- | --- | --- | --- | --- | --- |
|  | Median | Min. | Max. | Median | Min. | Max. |
| **Cytokines associated with** |  |  |  |  |  |  |
| **Innate Immunity** |  |  |  |  |  |  |
| **IL-1β** | 0.9 | 0.1 | 48.2 | 1.5 | 0.0 | 18.8 |
| **IL-6** | 11.0 | 1.0 | 469.2 | 7.7 | 0.1 | 47.4 |
| **TNFα** | 18.1 | 0.0 | 175.3 | 27.0 | 0.0 | 298.5 |
| **Adaptive Cellular Immunity** |  |  |  |  |  |  |
| **IL-12** | 17.0 | 1.9 | 2185.6 | 24.9 | 2.8 | 636.3 |
| **IFNγ^a^** | 41.8 | 9.0 | 216.7 | 51.5 | 5.7 | 626.5 |
| **IL-2** | 2.1 | 0.0 | 25.3 | 0.0 | 0.0 | 93.2 |
| **IL-15** | 2.8 | 0.0 | 22.0 | 0.0 | 0.0 | 19.7 |
| **IL-17** | 45.1 | 17.6 | 114.7 | 53.3 | 0.0 | 153.4 |
| **Adaptive Humoral Immunity** |  |  |  |  |  |  |
| **IL-4** | 5.6 | 1.6 | 12.7 | 5.6 | 1.0 | 10.2 |
| **IL-5^a^** | 3.0 | 0.5 | 68.4 | 4.2 | 0.2 | 69.1 |
| **IL-13** | 2.8 | 1.0 | 130.1 | 4.0 | 0.0 | 95.7 |
| **IL-9** | 13.6 | 0.0 | 2194.2 | 11.2 | 0.0 | 795.7 |
| **Immuno-suppression/ resolution** |  |  |  |  |  |  |
| **IL-1ra** | 139.3 | 66.0 | 441.5 | 126.6 | 41.1 | 663.3 |
| **IL-10** | 4.0 | 0.0 | 1008.4 | 2.7 | 0.0 | 373.3 |
| **Chemokines** |  |  |  |  |  |  |
| **Eotaxin** | 48.0 | 0.0 | 175.2 | 44.8 | 0.0 | 365.7 |
| **IL-8^a^** | 69.1 | 7.9 | 4635.7 | 28.6 | 4.1 | 141.5 |
| **IP-10^a^** | 1154.0 | 107.0 | 30319 | 749.9 | 177.7 | 7337.5 |
| **MCP-1** | 23.3 | 1.8 | 399.3 | 23.8 | 0.0 | 66.2 |
| **MIP-1α** | 4.6 | 0.0 | 80.1 | 3.9 | 0.0 | 27.6 |
| **MIP-1β^a^** | 90.2 | 15.6 | 473.8 | 72.5 | 15.2 | 223.4 |
| **RANTES** | 2941.8 | 1212.5 | 65180 | 2615.2 | 441.4 | 43545 |
| **Growth Factors** |  |  |  |  |  |  |
| **IL-7** | 7.1 | 1.9 | 165.0 | 10.4 | 0.7 | 103.2 |
| **FGF b^a^** | 13.8 | 0.0 | 67.9 | 21.5 | 0.0 | 164.5 |
| **G-CSF** | 19.9 | 8.3 | 365.6 | 17.8 | 2.7 | 118.7 |
| **GM-CSF^a^** | 0.0 | 0.0 | 86.7 | 0.0 | 0.0 | 23.6 |
| **PDGF-bb** | 4924.8 | 734.8 | 14319 | 3595.1 | 697.2 | 23350 |
| **VEGF^a^** | 75.6 | 10.6 | 620.8 | 42.3 | 3.1 | 136.6 |

**All results are pg/mL.** Values listed as zero are out of assay operating range.

**^a^** Comparisons of the difference between the onset distributions and 6-month follow-up distributions are statistically significant (p<0.05) by paired t-test or Wilcoxon signed rank test
